# Population pharmacokinetics of fedratinib in patients with myelofibrosis, polycythemia vera, and essential thrombocythemia

**DOI:** 10.1007/s00280-019-03929-9

**Published:** 2019-08-23

**Authors:** Ken Ogasawara, Simon Zhou, Gopal Krishna, Maria Palmisano, Yan Li

**Affiliations:** Translational Development and Clinical Pharmacology, Celgene Corporation, 556 Morris Ave, Summit, NJ 07901 USA

**Keywords:** Fedratinib, JAK2, Myelofibrosis, Polycythemia vera, Population pharmacokinetics

## Abstract

**Purpose:**

Fedratinib (SAR302503, TG101348) is an orally administered Janus kinase (JAK) 2-selective inhibitor that is being developed for the treatment of patients with myelofibrosis (MF). The objectives of this analysis were to develop a population pharmacokinetic (PK) model to characterize fedratinib concentration-time profiles in patients with MF, polycythemia vera (PV) and essential thrombocythemia (ET) following oral fedratinib administration; and to investigate the effects of selected covariates on fedratinib PK parameters.

**Methods:**

Nonlinear mixed effects modeling was employed in developing a population PK model for fedratinib. Intensive or sparse fedratinib concentration data collected in adult subjects with MF, PV or ET from six studies were pooled, and a total of 452 subjects and 3442 plasma concentration observations were included in the final model.

**Results:**

Fedratinib PK in patients with MF/PV/ET was adequately described by a two-compartment structural PK model with first-order absorption incorporating a lag time and first-order elimination. Following oral administration, fedratinib undergoes biphasic disposition and exhibits linear, time-invariant PK at doses of 200 mg and above. Compared to MF/ET patients, PV patients had higher apparent clearance (CL/F) and apparent central volume of distribution. Creatinine clearance was a statistically significant covariate on CL/F, and patients with mild and moderate renal impairment had 10% and 37% increases in fedratinib exposure as compared to patients with normal renal function. No clinically meaningful effect on fedratinib exposure was observed regarding age, body weight, sex, race and liver function.

**Conclusions:**

These results should serve as the basis for dose adjustment of fedratinib for special populations.

**Electronic supplementary material:**

The online version of this article (10.1007/s00280-019-03929-9) contains supplementary material, which is available to authorized users.

## Introduction

Myeloproliferative neoplasms (MPNs) are clonal, BCR-ABL1 negative hematopoietic diseases of myeloid proliferation, and characterized by abnormal production of terminally differentiated functional blood cells [1, 2]. MPNs are classically categorized into three disease entities: primary myelofibrosis (primary MF or PMF), polycythemia vera (PV) and essential thrombocythemia (ET) [1, 2]. Patients with PV and ET are characterized by an abnormal increase in hemoglobin/hematocrit and platelet count, respectively, and PMF is more advanced subtype of MPNs, associated with bone marrow fibrosis, release of profibrotic and proinflammatory cytokines and splenomegaly due to extramedullary hematopoiesis [3]. PV and ET may lead to secondary myelofibrosis (post-PV MF and post-ET MF, respectively) [1], which are clinically indistinguishable from PMF [4].

Janus kinase (JAK)/signal transducer and activation of transcription (STAT) pathway is key to cytokine receptor signaling and plays a critical role in hematopoiesis and immune response [5]. In human, the JAK family comprises four members: JAK1, JAK2, JAK3 and tyrosine kinase (TYK) 2, each of which associates with different cytokine receptors [6, 7]. Dysregulation of JAK-STAT pathway has been found in hematological malignancies and autoimmune diseases [5, 8, 9]. *JAK2* V617F, which induces constitutive activation of STATs, is identified in 95% of patients with PV and 50–60% of patients with PMF and ET, and this is the most prevalent mutation in MPNs [2].

Fedratinib (SAR302503, TG101348) is an oral kinase inhibitor with activity against wild type and mutationally activated JAK2 and FMS-like tyrosine kinase 3 (FLT3) that is being developed for the treatment of intermediate or high-risk primary or secondary (post-PV or post-ET) MF [10–13]. Fedratinib is selective for JAK2 over JAK1, JAK3 and TYK2, and inhibits wild-type JAK2, activated mutant *JAK2* V617F, and FLT3, with IC_50_ values of 3 nM, 3 nM and 15 nM, respectively [14]. Fedratinib significantly inhibits *JAK2* V617F-driven aberrant human PV progenitor erythroid differentiation [15]. Pharmacokinetics (PK) of fedratinib has been characterized in both healthy subjects [16, 17] and patients with MF [11, 13]. Fedratinib was rapidly absorbed following oral administration with peak plasma concentration attained within 0.5–4 h [11, 13]. Fedratinib exposure increased in an approximately dose-proportional manner over dose range of 300–500 mg at steady state [13]. Plasma fedratinib levels reached steady state within 15 days of once daily dosing, with mean accumulation ratio of 2.95–3.88 at 300–500 mg [13]. Mean terminal half-life of fedratinib was 62–78 h at the single dose of 300–680 mg in healthy subjects [17].

This article, to the best of our knowledge, for the first time describes a population PK model that was developed to characterize fedratinib concentration-time profiles in patients with MF, PV, or ET following oral fedratinib administration. In addition, the effects of covariates on fedratinib PK were investigated.

## Materials and methods

### Clinical study data

The population PK analysis utilized data from one phase 1 study (TED12037 [NCT00631462]), four phase 2 studies (ARD11936 [NCT01420770], ARD12042 [NCT01420783], ARD12181 [NCT01523171], ARD12888 [NCT01692366]) and one phase 3 study (EFC12153 [NCT01437787]). Study design, dosing regimen, and PK sampling information are presented in Supplementary Table 1. These studies were conducted in accordance with the Declaration of Helsinki and the International Council for Harmonisation Guideline for Good Clinical Practice (ICH E6). Written informed consent was obtained from all subjects.

### Bioanalytical methods

Concentrations of fedratinib in plasma were determined using a validated high-performance liquid chromatography with tandem mass spectrometric detection, with good accuracy (− 6.75–8.8%) and precision (4.84–13.11%). The lower limit of quantification was 1 ng/mL, and the calibration range was 1–1000 ng/mL.

### Population pharmacokinetic analyses

Population PK analyses were conducted using a nonlinear mixed-effect modeling approach, as implemented in the NONMEM software version 7.3.0 (ICON Development Solutions, Ellicott City, MD). Plotting of NONMEM outputs was conducted using the R software (version 3.4.1) and RStudio (version 1.1.456, Boston, MA).

In the development of the structural model, one- to three-compartment models with first-order elimination and different absorption models including first-order absorption with and without lag time, zero-order absorption and transit compartment model were tested to fit the plasma concentration-time data. First-order conditional estimation (FOCE) with interaction method was used for parameter estimation, with natural logarithm-transformed plasma fedratinib concentration data. The inter-subject variability was modeled assuming a log-normal distribution. Residual variability was modeled using an additive model. The model selection was based on the objective function value (OFV) using the log-likelihood ratio test, the goodness of fit criteria and visual predictive check (VPC). Covariate model building was carried out using a stepwise procedure, with significance levels set to 0.01 and 0.001 for the forward inclusion and backward elimination steps, respectively. Missing baseline covariates were imputed as the median value in the study population.

Stability of the final PK parameter estimates and the 95% confidence interval (CI) for the parameters were evaluated using the nonparametric bootstrap approach. With this approach, 500 datasets of size equal to the original dataset were generated by random resampling with replacement from the original dataset. The final model was fit to each of the 500 bootstrap datasets and all the model parameters were estimated for each dataset. The median and nonparametric 95% CI (2.5–97.5 percentiles) of the 500 estimates were calculated for each parameter. The ability of the final population PK model to describe the observed concentration data was evaluated by simulating 200 datasets having the same doses, dosing schedules and sampling times as the original dataset and by performing prediction-corrected VPC [18]. The 5th, 50th and 95th prediction percentiles of the fedratinib concentrations at each binned time point were computed for each simulated trial. Thereafter, the nonparametric 90% CI of the 5th, 50th and 95th prediction percentiles at each binned time point were calculated for the 200 simulated trials. The data were displayed graphically and overlaid with the corresponding percentiles of the observed data.

## Results

### Summary of analysis dataset

A total of 452 subjects with 3442 evaluable plasma fedratinib concentration records were included in the final population PK analysis dataset. Number of subjects and PK samples were summarized by the study in Supplementary Table 1. Demographic characteristics of these subjects are shown in Table 1. The subjects were primarily Caucasian (88%). They had a median (range) age of 65 (20, 95) years. The median (range) creatinine clearance (CLcr), a marker associated with renal function, was 78.5 (20.1, 181) mL/min. Based on National Cancer Institute Organ Dysfunction Working Group (NCI-ODWG) criteria, 115 mild and 17 moderate hepatic impairment patients were included in this analysis.

**Table 1:** Demographic and baseline characteristics of 452 patients with myelofibrosis, polycythemia vera or essential thrombocythemia

Characteristics		
Continuous variables	Median	(Range)
Age (year)	65	(20–95)
Weight (kg)	70.1	(39.5–135)
Creatinine clearance (mL/min)	78.5	(20.1–181)
Aspartate aminotransferase (U/L)	26	(6–174)
Alanine aminotransferase (U/L)	18	(2–159)
Total bilirubin (µmol/L)	12.0	(1.54–55.3)
Serum albumin (g/L)	41	(25–53)
Time since diagnosis (year)	3.46	(0–31.9)
Categorical variables	*N*	(%)
Sex		
Male	249	(55.1%)
Female	203	(44.9%)
Race		
Caucasian	399	(88.3%)
African-American	7	(1.5%)
Asian	44	(9.7%)
Others	2	(0.4%)
Ethnicity		
Hispanic or Latino	2	(0.4%)
Non-Hispanic or Latino	49	(10.8%)
Unknown	401	(88.7%)
Dose (mg)		
100	20	(4.4%)
120	3	(0.7%)
200	24	(5.3%)
240	3	(0.7%)
300	14	(3.1%)
360	3	(0.7%)
400	231	(51.1%)
500	108	(23.9%)
520	3	(0.7%)
600	3	(0.7%)
680	34	(7.5%)
800	6	(1.3%)
NCI-ODWG liver function classification		
Normal	320	(70.8%)
Mild	115	(25.4%)
Moderate	17	(3.8%)
ECOG		
0	174	(38.5%)
1	218	(48.2%)
2	57	(12.6%)
Unknown	3	(0.7%)
Diagnosis		
Primary MF	232	(51.3%)
Post-PV MF	91	(20.1%)
Post-ET MF	51	(11.3%)
PV	45	(10.0%)
ET	33	(7.3%)

*ECOG* Eastern Cooperative Oncology Group, *ET* essential thrombocythemia, *MF* myelofibrosis, *N* number of subjects, *NCI-ODWG* National Cancer Institute Organ Dysfunction Working Group, *PV* polycythemia vera

### Structural pharmacokinetic model characterization

To identify the structural model, a one-compartment PK model was compared with a two compartment PK model. The two-compartment model with first-order oral input was preferred over the one-compartment model with first-order oral input (∆OFV: − 1038). The two-compartment model was selected over the three-compartment model as the additional compartment did not improve the model fitting (no change in OFV). In addition, incorporating a lag time improved the model fitting by significantly decreasing OFV (∆OFV: − 37). The zero-order absorption model worsened the model fitting compared with the first-order absorption model (∆OFV: + 29). The transit compartment model was not pursued because the improvement of the goodness-of-fit plot and VPC were not observed compared to the model with lag time in spite of the improvement of OFV.

**Fig. 1:**
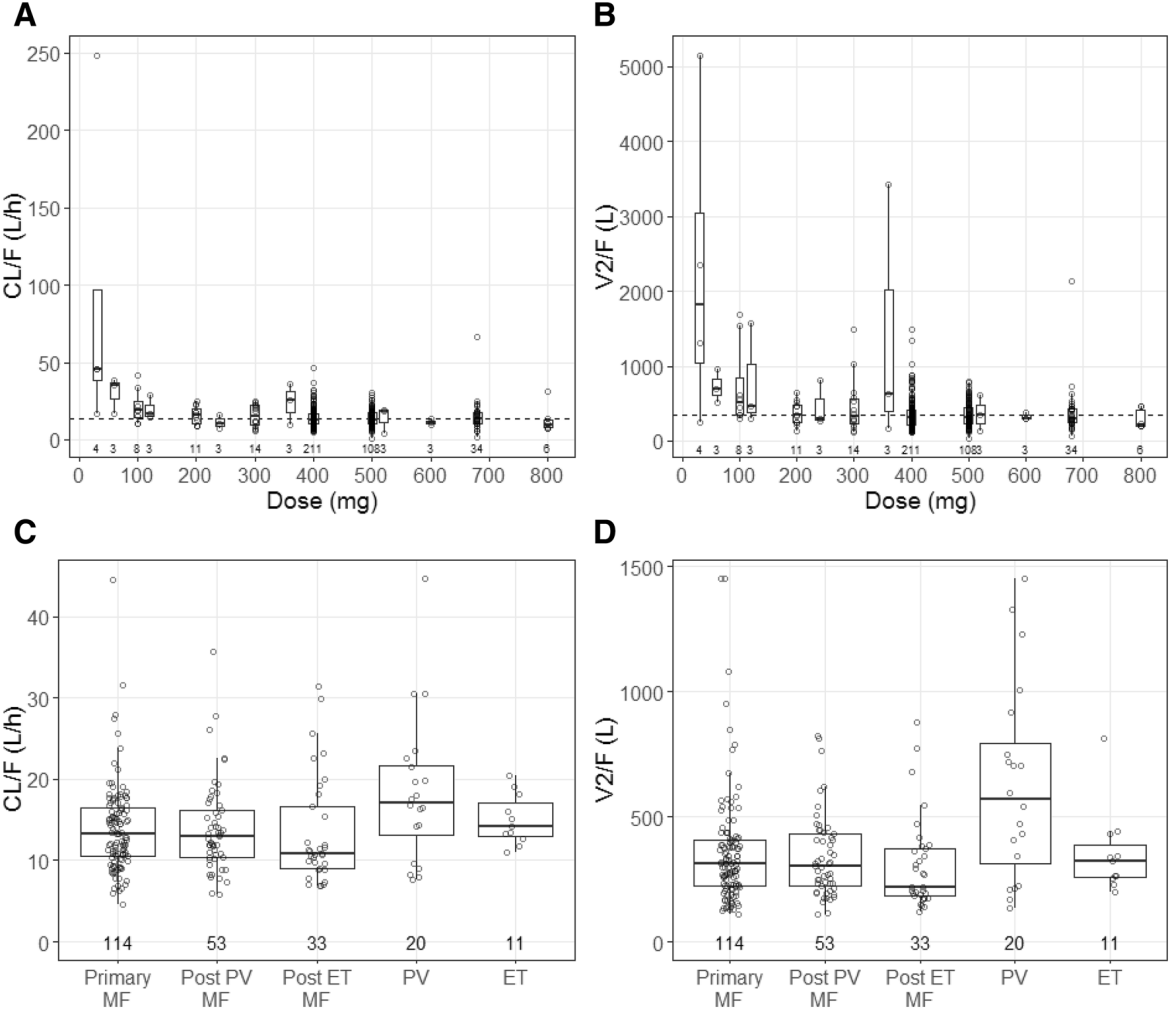
Box plot of apparent clearance (CL/F, **a** and **c**) and volume of distribution for central compartment (V2/F, **b** and **d**) of fedratinib by dose in patients with myelofibrosis and essential thrombocythemia (**a** and **b**) or by disease status in patients receiving 400 mg dose once daily (**c** and **d**). Individual estimates of CL/F and V2/F from the base model were overlaid on top of box plot, and the number of subjects at each dose level or disease status were listed below the box and whiskers. The dashed lines show typical values of CL/F (13.6 L/h) and V2/F plot (340 L) from the base model, respectively. *ET* essential thrombocythemia, *MF* myelofibrosis, *PV* polycythemia vera

Initial model exploration confirmed that apparent clearance (CL/F) values of fedratinib at doses of 100 mg and below were significantly higher than those at 200 mg and above (Fig. 1a). Apparent clearance appeared to be consistent and stable at doses of 200 mg and above, indicating that fedratinib PK is linear at doses of 200 mg and above. Similar finding was observed for apparent volume of distribution of central compartment (V2/F, Fig. 1b). Because there were limited PK data below 100 mg and the projected therapeutic dose in the target patient population was unlikely to be lower than 100 mg, the population PK analyses focused on clinically relevant doses at 100 mg and above after initial model exploration. According to goodness-of-fit and statistical criteria, a two-compartment model with first-order absorption rate constant (ka) incorporating a lag time, first-order elimination and error model adequately described fedratinib PK, and was selected as the final structural population PK model. Interindividual variability was determined for CL/F, V2/F, and ka.

Aside from doses, a visual examination of the dose-normalized concentration versus time profiles showed lower plasma fedratinib concentrations in PV patients than that in MF or ET patients. Individual CL/F and V2/F values from the base model confirmed that both CL/F and V2/F values were higher in PV patients than those in MF or ET patients (Fig. 1c and d). Therefore, the disease status of PV was subsequently added to both CL/F and V2/F as a categorical covariate in the covariate analysis.

### Final pharmacokinetic model

**Fig. 2:**
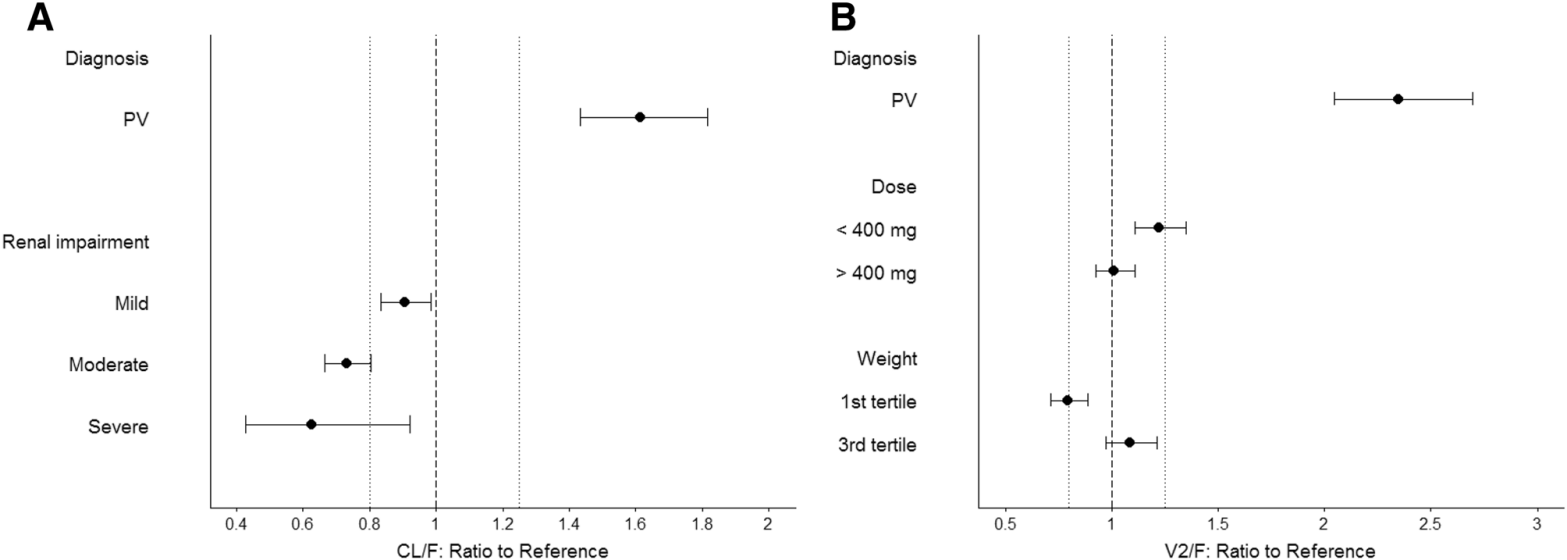
Forest plot of significant covariates on apparent clearance (CL/F, **a**) and volume of distribution for central compartment (V2/F, **b**) of fedratinib. Data are shown as median (90% confidence interval). References are myelofibrosis/essential thrombocythemia (diagnosis), normal renal function (creatinine clearance [CLcr] ≥ 90 mL/min), 400 mg (dose) and second tertile (weight). First, second and third tertile of weight at baseline are 39.5 to 64.6 kg, 65.0 to 76.8 kg and 77.0 to 135 kg, respectively. Mild 60 ≤ CLcr < 90 mL/min; moderate 30 ≤ CLcr < 60 mL/min; severe 15 ≤ CLcr < 30 mL/min

**Fig. 3:**
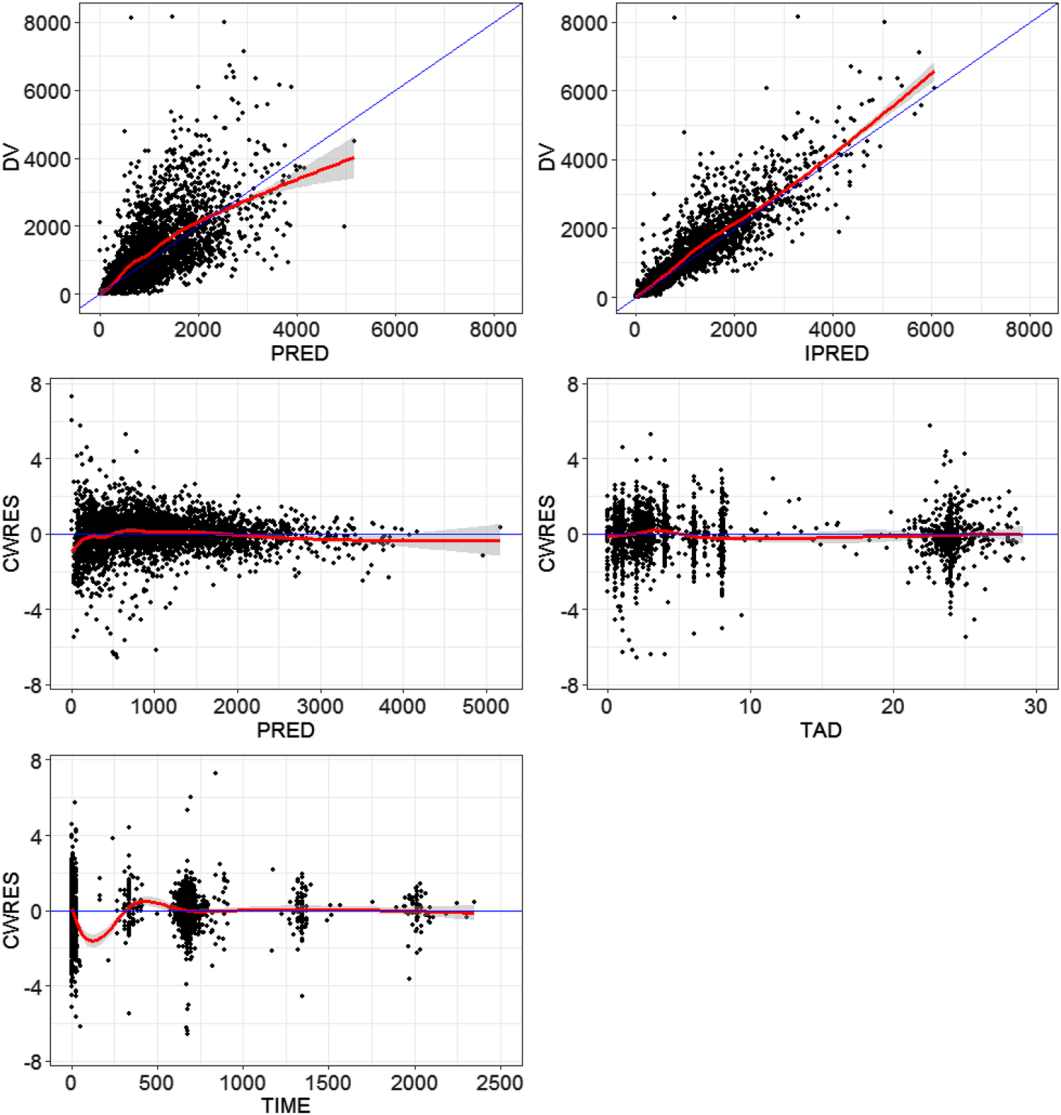
Goodness of fit plots of the final population pharmacokinetic model of fedratinib. The blue line represents the identity line or zero line. The red line represents the locally weighted scatterplot smoothing line. *CWRES* conditional weighted residuals, *DV* observed value, *IPRED* individual predicted values, *PRED* predicted values, *TAD* time after last dose (hour), *TIME* time after first dose (hour)

**Fig. 4:**
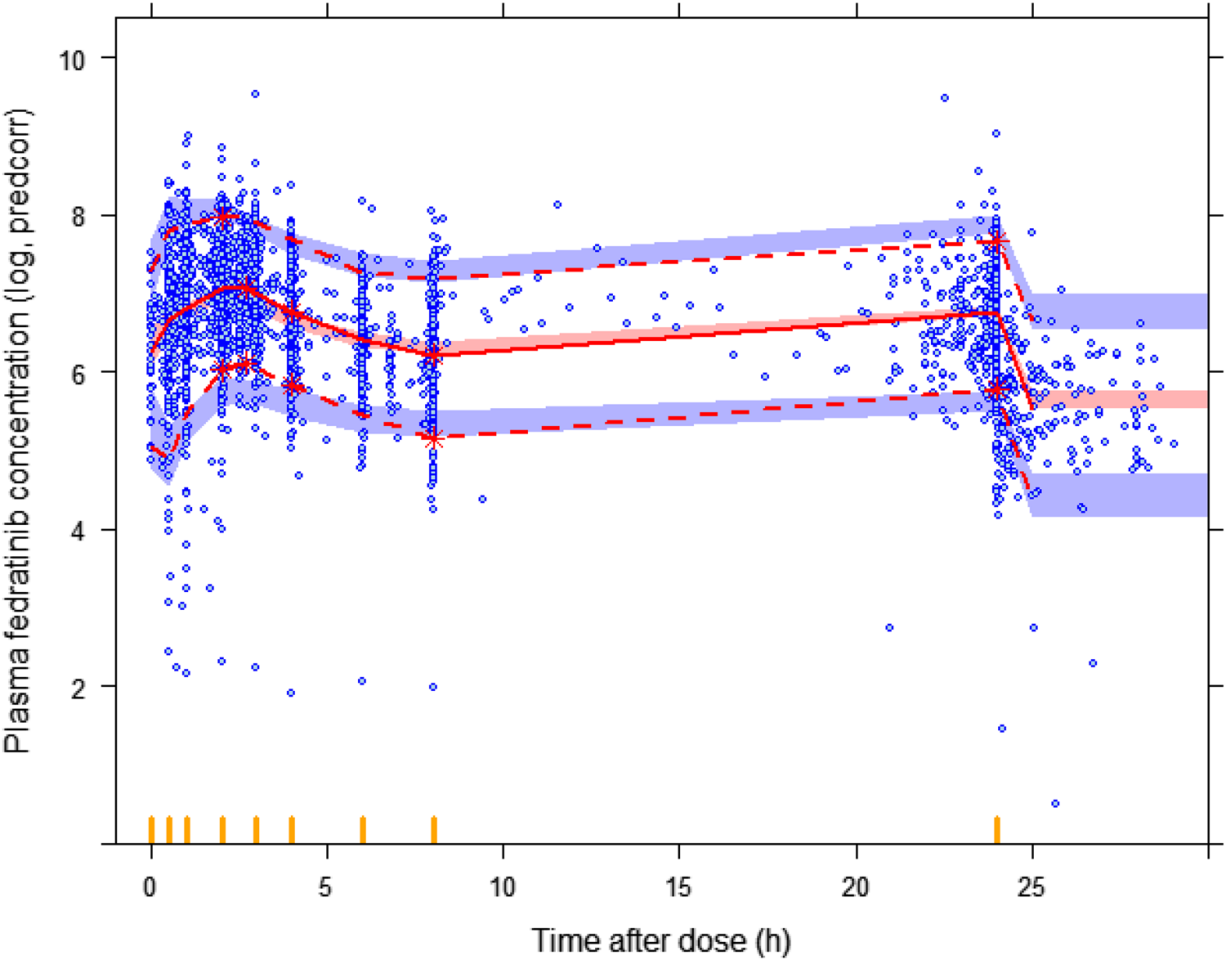
Prediction-corrected visual predictive check for plasma fedratinib concentration-time profiles. Circles represent observed data. Lines represent the 5th (dashed), 50th (solid), and 95th (dashed) percentiles of the observed data. Shaded areas represent nonparametric 90% confidence intervals about the 5th (blue), 50th (pink), and 95th (blue) percentiles for the corresponding model-predicted percentiles

Covariate model development using a stepwise procedure demonstrated that inclusion of PV on CL/F and V2/F, body weight and dose on V2/F and CLcr on CL/F significantly improved the model fitting. None of covariates were excluded from the final model during the backward elimination step. The PK parameters from the final population PK model for fedratinib are presented in Table 2. Most of the PK parameters for the final model were estimated with good precision (relatively narrow 95% CI from 500 bootstrap runs). The final model suggests that CL/F and V2/F was 54% and 87% higher, respectively, in PV patients compared to MF or ET patients, and patients with lower CLcr would have slightly lower CL/F of fedratinib (Fig. 2a). Due to the statistical significance of the effect of weight and dose on V2/F, the maximum plasma concentration (*C*_max_) of fedratinib would increase in slightly more than dose-proportional manner and would be inversely correlated to body weight (Fig. 2b). Goodness-of-fit plots indicated that the final model fitted well to the observed data (Fig. 3). Figure 4 presents the prediction-corrected VPC. There was a good agreement in the time course and central tendency between distributions of observed and simulated data, with no obvious bias. Overall, the estimated inter-individual variability adequately described the observed variability in plasma fedratinib concentrations.

**Table 2:** Population pharmacokinetic parameter estimates of fedratinib from the final model

Parameter	Parameter estimates	Bootstrap median (95% CI)^a^
Fixed effects		
TVCL/F (L/h)	13.0	13.1 (12.4 to 13.9)
TVV2/F (L)	311	313 (282 to 343)
TVQ/F (L/h)	45.2	45.2 (38.5 to 52.9)
TVV3/F (L)	1460	1470 (1190 to 1790)
TVKa (h^−1^)	1.57	1.64 (1.34 to 2.05)
TVALAG1 (h)	0.265	0.265 (0.264 to 0.321)
PV on CL/F^b^	1.54	1.51 (1.23 to 1.96)
CLcr on CL/F^b^	0.294	0.297 (0.157 to 0.441)
PV on V2/F^c^	1.87	1.85 (1.40 to 2.42)
Weight on V2/F^c^	0.727	0.733 (0.383 to 1.10)
Dose on V2/F^c^	− 0.279	− 0.279 (− 0.502 to − 0.0672)
Random effects		
Inter-individual variability		
*ω*^2^, CL/F	0.255	0.250 (0.176 to 0.352)
*ω*^2^, V2/F	0.383	0.371 (0.247 to 0.510)
COV_CL/F-V2/F_	0.197	0.193 (0.145 to 0.249)
*ω*^2^, Ka	1.07	1.09 (0.728 to 1.54)
Residual variability		
*σ*^2^ (Log additive)	0.201	0.194 (0.159 to 0.264)

*ALAG1* absorption lag time, *CI* confidence interval, *CL/F* apparent clearance, *COV* covariance, *CLcr* creatinine clearance, *Ka* absorption rate constant, *PV* polycythemia vera, *Q/F* apparent intercompartmental clearance, *TV* typical value, *V2/F* apparent volume of distribution of central compartment, *V3/F* apparent volume of distribution of peripheral compartment

^a^Bootstrap confidence interval values are taken from bootstrap calculation (484 successful out of a total of 500 bootstrap replicates)

^b^CL/F (L/h) = 13.0 * 1.54(if PV) * (CLcr/78.3)^0.294^

^c^V2/F (L) = 311 * 1.87(if PV) * (Weight/70.1)^0.727^ * (Dose/400)^− 0.279^

## Discussion

The fedratinib population PK model provided an adequate description of plasma fedratinib concentration-time data from MF, PV, or ET patients receiving oral fedratinib doses of 100 mg and above. Fedratinib concentration-time data were well characterized by the structural PK model that consists of a two-compartment with first-order absorption incorporating a lag time and first-order elimination.

Patients with PV had 87% higher apparent central volume of distribution and 54% higher apparent clearance compared to those in patients with MF or ET. Apparent clearance of a typical PV patient (CLcr = 78.5 mL/min) was 20.0 L/h, which falls into between CL/F of a typical MF patient (13.0 L/h) and CL/F of healthy subjects at doses of 300–680 mg (20.7–46.0 L/h) [11]. While patients with PV appear to have higher CL/F compared with patients with MF or ET, after progression to MF (post-PV MF), the CL/F appear to reach the similar level to that in patients with MF or ET. There were no apparent differences in CL/F and V2/F among primary MF, post-PV MF and post-ET MF, which is consistent with the clinical finding that both post-PV MF and post-ET MF are clinically indistinguishable from primary MF [4]. These results indicate that some MPN-related factor is associated with the reduced CL/F of fedratinib.

Patients with MF, PV, or ET appeared to have a comorbid condition of mild renal impairment as indicated by the lower CLcr (median 78.5 mL/min), although no patients with latent renal impairment were enrolled in the clinical studies. The renal function marker (CLcr) appeared to be statistically and positively correlated with CL/F of fedratinib, and the typical steady-state area under the concentration-time curve (AUC, dose divided by CL/F) for MF patients with mild (60 ≤ CLcr < 90 mL/min) and moderate (30 ≤ CLcr < 60 mL/min) renal impairment were 10% and 37% higher than that in MF patients with normal renal function (90 mL/min ≤ CLcr). Typical AUC for MF patients with severe renal impairment was 59% higher than that in MF patient with normal renal function, however, this should be interpreted with caution due to the small sample size (*N* = 3).

Fedratinib exhibited a greater than dose-proportional increase in exposure across a wide dose range in a phase 1 dose-escalation study in patients with MF (30–800 mg) [11] and an ascending single-dose study in healthy subjects (10–680 mg) [17]. In the base population PK model, the CL/F and V2/F were decreased with dose from 30 to 120 mg and remained dose-invariant at doses above 200 mg (Fig. 1a and b). The finding of more than dose-proportional increase of fedratinib exposure across wide dose range could be explained by larger distribution and/or elimination clearance at lower doses below 120 mg. Since the doses lower than 100 mg were deemed to be less efficacious and not studied beyond phase 2 studies, the final population PK modeling focused on the clinically relevant doses at 100 mg and above. In the covariate analysis, dose was statistically significant covariate on V2/F, however, the magnitude of changes in V2/F by dose was less than 30% and was considered not to be clinically meaningful. These results are consistent with the finding of a dose-proportional increase in fedratinib exposure over dose range of 300–500 mg at steady state [13].

Body weight (a range from 39.5 to 135 kg) was found to be statistically and positively correlated with V2/F. Large volume of distribution of fedratinib suggests that fedratinib may be distributed by diffusion into the extracellular fluids, the volume of which increases with body weight; thus, the estimated increases in the central volume of distribution of fedratinib with increased body weight are consistent with the physiological effects of weight. Given that the magnitude of changes in V2/F by body weight were less than 30%, and it does not affect AUC, body weight was deemed not to be a clinically relevant covariate.

In addition, no clinically meaningful effect on the PK of fedratinib was observed with regard to age (20 to 95 years), race, sex, mild hepatic impairment (defined as total bilirubin ≤ upper limit of normal [ULN] and aspartate aminotransferase [AST] > ULN or total bilirubin 1 to 1.5 times ULN and any AST) or moderate hepatic impairment (defined as total bilirubin > 1.5 to 3 times ULN and any AST), in the population PK analysis.

In summary, PK of fedratinib in patients with MF, PV, or ET was adequately described by a two-compartment model with first-order absorption incorporating a lag time and first-order elimination, and the fedratinib exposure increased linearly for doses 200 mg and above. The PV patients had 1.87-fold higher V2/F and 1.54-fold higher CL/F compared to that in MF or ET patients. Creatinine clearance was a statistically significant covariate on CL/F, and patients with mild and moderate renal impairment had 10% and 37% increase in fedratinib exposure, respectively, compared to patients with normal renal function. No clinically meaningful effect on fedratinib PK was observed with regard to other covariates such as body weight, age, race, sex, and mild and moderate hepatic impairment.

## Electronic supplementary material

Below is the link to the electronic supplementary material.

Supplementary material 1 (DOCX 15 kb)
