# Supplementary material for: Population pharmacokinetics of fedratinib in patients with myelofibrosis, polycythemia vera, and essential thrombocythemia
Source: Cancer Chemother Pharmacol. 2019 Aug 23;84(4):891–8. doi: 10.1007/s00280-019-03929-9 (PMC6768916; doi:10.1007/s00280-019-03929-9)
Supplement: Supplementary file 1 — Supplementary material 1 (DOCX 15 kb) [file 280_2019_3929_MOESM1_ESM.docx]

**Population Pharmacokinetics of Fedratinib in Patients with Myelofibrosis, Polycythemia Vera, and Essential Thrombocythemia**

Ken Ogasawara^1^, Simon Zhou^1^, Gopal Krishna^1^, Maria Palmisano^1^ and Yan Li^1^

1. Celgene Corporation, Summit, NJ

Correspondence: [yali@celgene.com](mailto:yali@celgene.com)

Cancer Chemotherapy and Pharmacology

**Supplementary Table 1. Summary of Clinical Studies Included in the Population Pharmacokinetic Modeling**

| **Study** | **Dosing regimen** | **Scheduled PK timepoints** | **Number of subjects in PopPK** | **Number of PK samples in PopPK** |
| --- | --- | --- | --- | --- |
| *TED12037 (MF-TG101348-001)*: A Phase 1, Open-label, Dose-escalation Study Evaluating the Safety, Tolerability, Pharmacokinetics, and Pharmacodynamics of Orally Administered TG101348 in Patients with Primary, Post-polycythemia Vera, or Post-essential Thrombocythemia Myelofibrosis | 30, 60, 120, 240, 360, 520, 680 and 800 mg QD | Cycle 1, Days 1 and 28: pre-dose and 0.5, 1, 2, 4, 8 and 28 h post-dose | 52 | 621 |
| *ARD11936*: A Phase 2 Randomized, Open-Label, Dose-Ranging Study of the Efficacy and Safety of Orally Administered SAR302503 in Patients with Intermediate-2 or High Risk Primary Myelofibrosis, Post-Polycythemia Vera Myelofibrosis, Post-Essential Thrombocythemia Myelofibrosis With Splenomegaly | 300, 400 and 500 mg QD | Cycle 1, Day 1: pre-dose and 1, 2, 3, 4, 6 and 8 h post-dose;  Cycle 1, Days 2 and 15: pre-dose;  Cycle 2, Day 1: pre-dose and 1, 2, 3, 4, 6 and 8 h post-dose;  Cycle 2, Day 2: pre-dose;  Cycle 3, Day 1: pre-dose | 31 | 516 |
| *ARD12042*: A Randomized Phase II, Open-Label Study of the Efficacy and Safety of Orally Administered SAR302503 in Patients with Polycythemia Vera (PV) or Essential Thrombocythemia (ET) Who Are Resistant or Intolerant to Hydroxyurea | 100, 200 and 400 mg QD | Cycle 1, Day 1: pre-dose and 1, 2, 3, 4, 6 and 8 h post-dose;  Cycle 1, Days 2 and 15: pre-dose;  Cycle 2, Day 1: pre-dose and 1, 2, 3, 4, 6 and 8 h post-dose;  Cycle 2, Day 2: pre-dose;  Cycle 3, Day 1: pre-dose | 78 | 1206 |
| *ARD12181*: A Phase II, Multicenter, Open Label, Single Arm Study of SAR302503 in Subjects Previously Treated with Ruxolitinib and With a Current Diagnosis of Intermediate or High-Risk Primary Myelofibrosis, Post-Polycythemia Vera Myelofibrosis, or Post-Essential Thrombocythemia Myelofibrosis | 400 mg QD | Cycles 1 and 2, Day 1: pre-dose and 0.5-2 and 2.5-4 h post-dose;  Cycle 4, Day 1: pre-dose | 97 | 511 |
| *ARD12188*: A Phase 2 Open-Label, Dose-Ranging Study of the Efficacy and Safety of Orally Administered SAR302503 in Japanese Patients with Intermediate-2 or High Risk Primary Myelofibrosis, Post-Polycythemia Vera Myelofibrosis, Post-Essential Thrombocythemia Myelofibrosis with Splenomegaly | 300, 400 and 500 mg QD | Cycle 1, Day 1: pre-dose and 1, 2, 3, 4 and 8 h post-dose;  Cycle 1, Days 2, 8 and 15: pre-dose;  Cycle 2, Day 1: pre-dose and 1, 2, 3, 4 and 8 h post-dose;  Cycle 2, Day 2: pre-dose;  Cycle 3, Day 1: pre-dose | 8 | 97 |
| *EFC12153*: A Phase 3, Multicenter, Randomized, Double-Blind, Placebo-Controlled, 3-Arm Study of SAR302503 in Patients with Intermediate-2 or High-Risk Primary Myelofibrosis, Post-Polycythemia Vera Myelofibrosis, or Post-Essential Thrombocythemia Myelofibrosis with Splenomegaly | 400, 500mg and placebo QD | Cycles 1 and 2, Day 1: pre-dose and up to 4 h post-dose | 186 | 491 |

PopPK, population pharmacokinetics; QD, once daily; SAR302503, fedratinib; TG101348, fedratinib
